# Swedish abdominal massage against warm water therapy on postoperative orthopaedic surgery constipation: a comparison quasi-experimental study

**DOI:** 10.12688/f1000research.159217.3

**Published:** 2025-10-03

**Authors:** Enny Selawaty Boangmanalu, Masfuri Masfuri, Muhamad Adam, Sri Nining, Triani Banna, Indira Mastura Pulungan

**Affiliations:** 1 Faculty of Nursing, Universitas Indonesia, Depok, West Java, 16424, Indonesia; 2 Faculty of Nursing, Universitas Kristen Krida Wacana, West Jakarta, Jakarta, 12120, Indonesia; 3 Nursing Committee, Mother and Child Bunda Jakarta Hospital, Menteng, Central Jakarta, 10350, Indonesia; 4 Nursing Science, Sekolah Tinggi Ilmu Kesehatan Papua, Sorong City, West Papua, 98412, Indonesia

**Keywords:** Closed Fracture Reduction, Constipation, Postoperative, Swedish Abdominal Massage, Warm Water Therapy

## Abstract

**Background:**

Postoperative immobilization in patients with lower extremity fractures frequently leads to constipation, affecting approximately 50–70% of patients. Non-pharmacological nursing interventions such as Swedish abdominal massage and warm water drinking therapy are potential approaches to alleviate this problem.

**Objective:**

The objective of this study was to compare the effectiveness of Swedish abdominal massage and warm water drinking therapy in reducing constipation scores among patients with postoperative lower extremity fractures.

**Methods:**

A quasi-experimental pre–posttest design without a control group was conducted with 30 respondents selected using a simple random sampling technique. Constipation was assessed using the Constipation Assessment Scale (CAS) before and after intervention. Data were analyzed using independent *t*-tests, and results were interpreted with attention to baseline differences between groups.

**Results:**

Both interventions significantly reduced constipation scores. The mean post-intervention CAS score was 4.60 in the warm water group and 3.56 in the Swedish abdominal massage group ( *p* < 0.001). Although Swedish abdominal massage showed a greater within-group reduction, baseline imbalances in constipation scores may have influenced the observed differences.

**Conclusion:**

Swedish abdominal massage and warm water therapy are effective nurse-led, non-pharmacological interventions for reducing postoperative constipation. The findings should be interpreted cautiously due to baseline differences and small sample size. Future research with larger samples, controlled designs, and statistical adjustments is recommended to confirm these preliminary findings.

## Background

According to the most recent data published in Lancet Healthy Longev, there were 178 million new fractures globally in 2019, and 455 million people had acute or chronic fracture symptoms at some point in their lives ( Global Burden of Disease, 2021). However after surgery, prolonged bed rest and limited movement inevitably result in adverse effects such as constipation ( Viberg et al., 2022). Constipation, which typically affects 50–70% of patients, is brought on by the body’s immobile state following surgery, which weakens intestinal peristalsis function in conjunction with dietary factors ( Jing & Jia, 2019). Patients should be incredibly concerned about constipation because it can lead to a number of issues and negatively impact their quality of life ( Kamali et al., 2022). Consequently, constipation is a serious problem that, if disregarded, can result in psychological and physical problems. Laxative use is common among patients due to discomfort. Research shows that constipation affects 3% to 27% of people in the general population on an incidence rate of 5; in hospital settings, the prevalence is 79% ( Pehlivan & Nural, 2022). However, in acute clinical practice, this condition is frequently overlooked when providing patient care ( Trads et al., 2018).

Nurses have been treating constipation with a nursing intervention that includes teaching patients about high-fiber foods like vegetables and papaya ( Galica et al., 2022). But there has never been an application of another autonomous nursing intervention in the treatment room, like abdominal massage ( Nouhi et al., 2022). It is relatively inexpensive and something that sufferers can do on their own. Constant direct pressure is applied to the abdominal wall during abdominal massage, which is followed by a relaxation period. This increases the contractions of the rectum and intestines and the gastrocolic reflex ( Yao et al., 2020). Swedish abdominal massages are performed with light pressure on the tissue to promote comfort and enhance the digestive and blood circulation systems ( Park et al., 2023). By altering stomach pressure through mechanical and reflexive processes, abdominal massage can accelerate the passage of food through the digestive system and promote peristaltic movements ( Fekri et al., 2021). This procedure will speed up the movement of food through the digestive tract by increasing peristaltic movements and altering stomach pressure through mechanical and reflexive means ( Lafcı & Kaşikçi, 2023).

To fill the stomach capacity, water is a good option. Using warm water as a complementary therapy is recommended ( Kilroe et al., 2024). The effects of hydrostatic, hydrodynamic, and warmth can promote relaxation and better blood circulation. The body responds to water to prevent, correct, and improve human health status, which makes water therapy one of the natural healing systems ( Asmaa Sayed et al., 2018). Warm water consumption will quicken the body’s temperature regulation process because it requires less energy. Constipation will speed up the defecation process by using warm water to soften the stool ( Parsons et al., 2022). The aim of this study is to compare the effectiveness of drinking warm water and Swedish abdominal massage on constipation scores on post-operative lower extremity fractures.

## Methods

### Study design

This research used quasi-experimental and pre-posttest designs without control group. This research was conducted at the Idaman Regional Public Hospital, Banjarbaru, South Kalimantan, Indonesia from May 10 ^th^ to June 30 ^th^ 2023.

### Participants

The sample was 30 respondents selected using simple random technique. Determination of the sample size of each group is determined by calculating the paired numerical comparative formula. The sample in this research selected randomly using a lottery. Randomization is done by lottery, which makes a list of all subjects to be studied, gives a number code to each item to be investigated, writes the code on a small paper, rolls up each paper, puts the rolled paper into a container then shakes or shakes the container and takes one by one the roll. If you get an odd number, the warm water consumption intervention will be carried out while if you get an even number, you will enter the Swedish abdominal massage intervention. The inclusion criteria were: (1) all post-operative orthopedic surgery patients with lower extremity fractures (fractures of the femur, pelvis, tibia, fibula, ankle, and pedis), (2) patients with casts, (3) post-operative orthopedic surgery patients with ORIF, (4) patients aged 17-60 years, (5) compos-mentis patient, (5) patients with post-operative day 1 (after 1×24 hours/POD 1), (6) patients with a Barthel index score ≤ 8 (severe dependence), (7) patients receiving a standard diet from the hospital.

### Instrument

This research used demographic questionnaire and the Constipation Assessment Scale (CAS) questionnaire to assess the patients’ constipation levels before and after the intervention. We used the Constipation Assessment Scale (CAS) that develop by McMillan and Williams and translated into Indonesian version by Suwandi. The valid CAS instrument in the Indonesian version consists of eight questions ( Suwandi, 2017).

### Intervention

The sample was split into two intervention groups: group 2 got a Swedish abdominal massage, and group 1 drank warm water. 500 ml of warm water in a glass was consumed by group 1 at a temperature of 31.5°C. For three days, they drank the water as soon as they woke up and before breakfast. They were then permitted to eat breakfast 30 to 45 minutes after they had finished drinking. Over the course of three days, the second group, which was given a Swedish abdominal massage, had their abdominal muscles massaged for fifteen to twenty minutes. The moment when the respondents woke up, they were received this massage. It is recommended that patients refrain from eating breakfast following the intervention; however, they are permitted to consume food 30 to 45 minutes following the procedure. Every intervention was examined over the course of three days; the pretest was given prior to the intervention, and the post test was given following the third day of the intervention. The first time the respondent was able to defecate was used to calculate the defecation time.

### Data collection

The data were collected by the researchers. Questionnaires were distributed to each group before and after intervention.

### Data analysis

Descriptive statistical tests were used to measure demographic data or respondents’ characteristics. Data analysis was done with Windows-based IBM SPSS (Version 26.0) ( IBM Corp, 2024). The data were normally distributed according to Shapiro-Wilk (p≥0.05), so the independent t-test was used to measure constipation scores. The data were normally distributed according to Shapiro-Wilk (p≥0.05), so the independent t-test was used to measure constipation scores.

## Results

The participants’ characteristics such as age, gender, types of analgetics and constipation score were collected at baseline on Table 1.

**Table 1.** Respondent’s characteristics.

Characteristics		Warm water drinking therapy (n = 15)		Swedish abdominal massage (n = 15)		Total (n = 30)	
		n	%	n	%	n	%
**Sex**							
Female		4	26.66%	6	40%	10	33.3%
Male		11	73.33%	9	60%	20	66.6%
**Age**		Mean (SD) 28.93 (14.97)		Mean (SD) 31.53 (16.16)			
**Types of analgetic**							
**Non-opioid **		15	100%	15	100%	30	100%
**Constipation Score**	Before	Mean (SD) 8.40 (2.41)		Mean (SD) 10.13 (1.80)			
	After	Mean (SD) 4.60 (0.91)		Mean (SD) 3.60 (0.63)			

According to this study, the majority of respondents—11 (73.3%) and nine (60%), respectively—were men who participated in the warm water intervention group and the Swedish abdominal massage intervention group. The first intervention group’s average age was 28.93 years, while the second intervention group’s average age was 31.53 years. The first intervention group’s confidence interval fell between 17 and 60 years, while the second intervention group’s confidence interval fell between 17 and 58 years. In both groups, non-opioid analgesics were used 100% of the time as analgesics. Prior to the intervention, the group receiving a Swedish abdominal massage had a higher mean constipation score than the group drinking warm water. On the other hand, the Swedish abdominal massage intervention group had a lower mean constipation score following the intervention. The statistical test found significant differences between the two intervention groups’ respondent characteristics (gender, age, and type of analgesic; p>0.05), but not between the groups’ pre- and post-intervention characteristics.

### Constipation scores of the warm water drinking therapy group and the swedish abdominal massage group

The difference in constipation score was measured by looking at the mean score of the drinking warm water group and the Swedish abdominal massage group on Table 2.

**Table 2.** Constipation scores of the warm water drinking therapy group (n = 15) and the swedish abdominal massage group (n = 15).

Group	Pre-test Mean (SD)	Post-test Mean (SD)	Mean Difference	95% CI of Difference	p-value	Effect Size (Cohen’s d)
Warm water drinking therapy	8.40 (2.41)	4.60 (0.91)	3.80	[2.50 – 5.10]	<0.001	2.09
Swedish abdominal massage	10.13 (1.80)	3.60 (0.63)	6.53	[5.56 – 7.50]	<0.001	4.84

* Significant at p<0.05.

The within-group analysis demonstrated that both interventions significantly reduced constipation scores. In the warm water group, the mean CAS score decreased from 8.40 (SD = 2.41) to 4.60 (SD = 0.91), *t*(14) = xx, *p* < 0.001, Cohen’s *d* = 2.09, 95% CI [2.50–5.10]. Similarly, the Swedish abdominal massage group showed a reduction from 10.13 (SD = 1.80) to 3.60 (SD = 0.63), *t*(14) = xx, *p* < 0.001, Cohen’s *d* = 4.84, 95% CI [5.56–7.50].

Between-group comparisons indicated a greater mean reduction in constipation scores in the massage group (mean difference = 6.53, 95% CI [5.56–7.50]) compared to the warm water group (mean difference = 3.80, 95% CI [2.50–5.10]). However, the baseline imbalance in constipation scores (10.13 vs. 8.40) may have influenced these results, and therefore the interpretation of superiority between groups should be made cautiously.

## Discussion

Both Swedish abdominal massage and warm water therapy were effective in reducing postoperative constipation scores. Within-group analyses confirmed significant reductions for both interventions, each with large effect sizes. Although the Swedish abdominal massage group demonstrated a greater mean reduction, this finding should be interpreted with caution because the baseline constipation scores were higher in this group. The imbalance at baseline may have provided a greater potential for improvement, and the small sample size further limits the strength of between-group comparisons.

In addition to being an effective treatment for constipation, a Swedish abdominal massage can also lessen the degree of straining, anal pain, and bloating, as well as the degree of incomplete bowel emptying ( Choi et al., 2021). By strengthening the abdominal muscles and encouraging intestinal peristalsis, massage can improve the digestive system’s efficiency, improving quality of life ratings and resulting in better-consistent stools ( Durmuş İskender & Çalışkan, 2022). Abdominal massage can stimulate afferent neuron stretch receptors in the luminal wall, which contract the intestinal muscles above the pressure point and relax them below, thereby generating peristaltic waves ( Aydinli & Karadağ, 2023; Faghihi et al., 2021; Keely & Barrett, 2022). This physiological mechanism supports the observed clinical improvements.

For three days in a row, consuming 500 ml of warm water first thing in the morning can intensify the gastric effect and heighten the feeling of passing gas ( Kilroe et al., 2024). The best time to trigger the gastrocolic reflex is in the morning. This reflex happens when the extrinsic autonomic nerve, which is responsible for promoting colonic motility and large amplitude propagation (HAPCs) to ward off constipation, contracts the stomach when it reaches a specific volume (500 ml) ( Al-Kharraz et al., 2023). One to three glasses of water a day can help trigger the gastric reflex. Utilizing warm water is a complementary therapy. The warm, hydrostatic, and hydrodynamic effects can promote relaxation and better blood circulation ( Asmaa Sayed et al., 2018).

Significant results were found in the majority of research studies that used warm water consumption at different volumes in conjunction with Swedish abdominal massage. The hypothesis posits that upon food entry, the large intestine experiences mass movements primarily due to the gastrocolic reflex ( Bellini et al., 2021). This reflex is facilitated by gastrin and parasympathetic innervation, which travel from the stomach to the large intestine ( Chatip et al., 2024). The urge to urinate frequently follows this reflex, which is most noticeable in many people after their first meal of the day. Therefore, a reflex is triggered to move the contents of the digestive tract farther along when new food enters, making room for the incoming food ( Gu et al., 2023). The remaining small intestine’s contents are moved into the large intestine by the gastroileal reflex, and the defecation reflex is triggered by the gastrocolic reflex, which pushes the large intestine’s contents into the rectum. This is so that the digestive system can function more efficiently. Peristalsis, or the stimulation of intestinal peristaltic movements, is strengthened by abdominal massage and is consistent with the findings of previous studies ( Karaaslan et al., 2024).

Previous studies have also described the physiological mechanisms underlying these effects. With the help of gastrin from the stomach and the extrinsic autonomic nerve, direct stimulation of the abdominal muscles can induce peristalsis and the gastrocolon, which causes the colon’s mass to accelerate and the stomach’s contractions to strongen ( Chatip et al., 2024). A gastroileal reflex will result in the movement of the remaining contents of the small intestine to the large intestine by forcing the chyme into the duodenum through strong peristaltic movements ( Durmuş İskender & Çalışkan, 2022). This will speed up the absorption process in the intestine. A colon’s contents will be forced into the rectum, a gastrocolon effect will be produced, stretch receptors in the rectal wall will be stimulated, and a feeling of defecation will be produced through good intestinal motility ( Artale et al., 2023). These mechanisms help to explain the observed improvements in constipation scores in both intervention groups, although our study was not designed to measure physiological parameters directly.

Overall, both interventions are feasible nurse-led strategies, but the findings must be interpreted with caution. The small sample size (n = 30), the absence of power analysis, and baseline imbalance are important limitations that restrict generalizability. Future studies should employ larger samples, rigorous randomization, and appropriate statistical adjustments such as ANCOVA to minimize baseline differences and confirm these preliminary findings.

## Conclusion

Both Swedish abdominal massage and warm water therapy significantly improved constipation symptoms in postoperative patients with lower extremity fractures. Swedish abdominal massage showed a greater reduction within its group; however, this result should be interpreted with caution due to baseline differences, the small sample size, and other contextual limitations. Despite respondents following hospital dietary guidelines, fiber intake and food portions were not calculated, which may have influenced outcomes. Furthermore, fracture location may have affected mobilization, with distal fractures allowing easier mobilization compared to proximal fractures.

Overall, both interventions may be considered as feasible, nurse-led strategies to complement pharmacological management of postoperative constipation. Larger, adequately powered studies with longer follow-up and more comprehensive control of confounding factors (such as diet and mobilization) are warranted to confirm these findings and guide evidence-based postoperative care protocols.

## Ethical consideration

This research was approved by the Ethics Commission of Idaman Regional Public Hospital, Banjarbaru with number No. RS00214/KEPK-RSDI/04/2023 on May 1 ^st^ 2023. Before conducting the research, the researcher explained to the respondents about the objectives, procedures, and expectations of this research in person verbal and written. The respondents were asked to provide their written consent in an informed consent by signing before participating in this research. The respondents were also assured that their involvement was voluntary and were informed of their right to withdraw from the study at any time without facing any penalties.

## Data Availability

Fighshare: Swedish Abdominal Massage versus Warm Water Therapy on Postoperative Constipation: A Comparison Quasi Experimental Study: 10.6084/m9.figshare.27764655.v2 ( Boangmanalu, 2024). This project contains the following underlying data Click or tap here to enter text.
1. SPO of Water Consumption 2. SPO of Abdominal Massage 3. SPSS Output 4. All Data consist of all raw data underlying data before analysis in SPSS file Data are available under the terms of the Creative Commons Attribution 4.0 International license (CC-BY 4.0). Fighshare: Swedish Abdominal Massage versus Warm Water Therapy on Postoperative Constipation: A Comparison Quasi Experimental Study: 10.6084/m9.figshare.27764655.v2 ( Boangmanalu, 2024). This project contains the following extended dataClick or tap here to enter text.:
1. Consort cheklist 2. Research instrument 3. Informed Consent 4. Research explanation Data are available under the terms of the Creative Commons Attribution 4.0 International license (CC-BY 4.0). Fighshare: CONSORT checklist for Swedish Abdominal Massage versus Warm Water Therapy on Postoperative Constipation: A Comparison Quasi Experimental Study: 10.6084/m9.figshare.27764655.v2 ( Boangmanalu, 2024). Data are available under the terms of the Creative Commons Attribution 4.0 International license (CC-BY 4.0).
