# Supplementary material for: Swedish abdominal massage against warm water therapy on postoperative orthopaedic surgery constipation: a comparison quasi-experimental study
Source: F1000Res. 2025 Oct 3;13:1531. Originally published 2024 Dec 18. [Version 3] doi: 10.12688/f1000research.159217.3 (PMC12340488; doi:10.12688/f1000research.159217.3)
Supplement: Peer review history

# Reviewer report: Reviewer response for version 2

**DOI:** 10.5256/f1000research.183692.r395120

**Published:** 2025-08-11

**Authors:** Jie Hao

**Affiliations:** 1 University of Nebraska Medical Center, Omaha, USA

The manuscript entitled "Swedish abdominal massage against warm water therapy on postoperative orthopaedic surgery constipation: a comparison quasi-experimental study" has been reviewed. The manuscript explores a relevant and practical nursing intervention—Swedish abdominal massage versus warm water therapy—for managing postoperative constipation in patients with lower extremity fractures. The topic is of clinical interest, particularly given the high incidence of postoperative constipation and the need for non-pharmacological, nurse-led interventions.

 The baseline constipation scores between the two intervention groups differ notably (10.13 vs. 8.40), raising concerns about the comparability of groups. While the authors mention no significant differences in demographics (p>0.05), the baseline CAS differences affect the internal validity of between-group comparisons.

 The conclusion that "Swedish abdominal massage was noticeably more successful" may be overstated given this imbalance. A within-group pre-post comparison would be more appropriate, or statistical adjustment could help account for baseline differences.

 The study includes only 15 participants per group, which limits the power to detect meaningful differences and generalize the findings. No power analysis is provided to justify the sample size, and effect sizes are not reported.

 The results report p-values but not confidence intervals or effect sizes, which are essential for interpreting clinical significance.

Is the work clearly and accurately presented and does it cite the current literature?

Yes

If applicable, is the statistical analysis and its interpretation appropriate?

No

Are all the source data underlying the results available to ensure full reproducibility?

Yes

Is the study design appropriate and is the work technically sound?

Yes

Are the conclusions drawn adequately supported by the results?

No

Are sufficient details of methods and analysis provided to allow replication by others?

No

Reviewer Expertise:

Physical therapy, rehabilitation

I confirm that I have read this submission and believe that I have an appropriate level of expertise to confirm that it is of an acceptable scientific standard, however I have significant reservations, as outlined above.

# Response

**Published:** 2025-09-24

**Authors:** Sri Nining

**Affiliations:** Nursing Committee, Woman and Children Bunda Jakarta, Central Jakarta, akarta, Indonesia

**Comment 1**

*The baseline constipation scores between the two intervention groups differ notably (10.13 vs. 8.40), raising concerns about the comparability of groups. While the authors mention no significant differences in demographics (p>0.05), the baseline CAS differences affect the internal validity of between-group comparisons.*

**Response:**

 Thank you for pointing this out. We have revised the *Discussion* section to highlight this limitation. We now explicitly state that the baseline imbalance may have influenced the results, and that the observed superiority of Swedish abdominal massage should be interpreted cautiously. In addition, within-group pre-post analyses were added to the *Results* section to provide a clearer picture of intervention effectiveness. We also recommend the use of ANCOVA or stratified randomization in future studies to minimize baseline variability.

**Comment 2**

*The conclusion that “Swedish abdominal massage was noticeably more successful” may be overstated given this imbalance. A within-group pre-post comparison would be more appropriate, or statistical adjustment could help account for baseline differences.*

**Response:**

 We agree with this comment. The statement in the *Discussion* and *Conclusion* sections has been revised to a more cautious interpretation. We now emphasize that both interventions were effective, with Swedish abdominal massage showing greater within-group reduction, but superiority cannot be firmly concluded due to baseline differences and the small sample size.

**Comment 3**

*The study includes only 15 participants per group, which limits the power to detect meaningful differences and generalize the findings. No power analysis is provided to justify the sample size, and effect sizes are not reported.*

**Response:**

 We have acknowledged the small sample size as a key limitation in the *Discussion* section and clarified that it reduces statistical power and generalizability. To strengthen the findings, we have now reported effect sizes (Cohen’s *d*) and 95% confidence intervals in the *Results* section and revised Table 2 accordingly. These additions provide greater clarity regarding the clinical significance of the results.

**Comment 4**

*The results report p-values but not confidence intervals or effect sizes, which are essential for interpreting clinical significance.*

**Response:**

 This has been addressed in the revised *Results* section and Table 2, where we now include 95% confidence intervals and effect sizes for both interventions. The narrative has also been updated to interpret these findings more appropriately.

# Reviewer report: Reviewer response for version 2

**DOI:** 10.5256/f1000research.183692.r395119

**Published:** 2025-08-09

**Authors:** Emil Huriani

**Affiliations:** 1 Faculty of Nursing, Universitas Andalas, Padang, West Sumatra, Indonesia

Thank you for the opportunity to review this manuscript.

 Overall, this is a well-conducted study. There are several extents need to be executed to make the conclusion of the study is readable and acceptable.

**Background**

 Background does not explain well the magnitude of the problem, the urgency of the research, and the state of the art based on the available literature.

 The researcher does not reveal the novelty of the research. The application of abdominal massage to overcome constipation has been widely documented in references. The author has not explained the advancement revealed through this research.

 I'm a little confused because in the background of the study, the researchers stated that an intervention involving "teaching patients about high-fiber foods like vegetables and papaya" could relieve constipation. In contrast, the researchers tested the effect of using warm water therapy to relieve constipation.

 Another confusing statement is "Constipation will speed up the defecation process by using warm water to soften the stool.". What do you mean with this statement?

 The authors need to explain why they chose to compare abdominal massage and warm water therapy in this study.

**Methods**

**Participants**

 Regarding randomization, researchers do not need to explain the lottery process in detail. Were there any exclusion criteria for selecting respondents? Please provide these.

 Was there any control over the type of anesthesia used, whether spinal or general?

**Instruments**

 What is the validity and reliability of the CAS score after being translated into a bahasa?

 In intervention section, it is stated “Over the course of three days, the second group, which was given a Swedish abdominal massage, ‘had their abdominal muscles massaged for fifteen to twenty minutes.” Then, what was done during the first three days.

 Researchers recorded the first time the respondent was able to defecate to calculate the defecation time. I couldn't find this data in the results table.

**Data Analysis**

 There is a repetition of sentences at the end of this section.

**Ethical approval**

 It's important to explain the ethics protocol and how researchers meet the ethical aspects of health research. Has the research protocol received ethical approval from the appropriate agency?

**Results**

 Researchers need to provide data on laxative use because laxative use is often used to treat constipation and the Barthel index score because this score describes the level of dependency and the level of ability to carry out activities.

 Before continuing to statistical test on differences of CAS score after the intervention between the two respondents’ groups, authors must go first with homogeneity test of CAS score before the intervention between the two respondents’ groups as well as homogeneity tests for respondent characteristics (age and gender). When the CAS scores before the intervention in both groups were found to be homogeneous, then the statistical test can be continued by comparing the CAS scores after the intervention. Otherwise, if the data at the baseline were not homogeneous, the researcher should go with a statistical test that was appropriate for non-homogeneous data.

 The research results are not in sync with the SPSS output in the Extended data related to participants’ characteristics such as age and gender. The extended data provided shows repetitions on analysis of research data and the results are different between the initial analysis and the subsequent analysis.

 In Table 2 dan its narration, it is confusing when you are presenting the difference constipation score, but still there are constipation scores both pre-test and post-test of both groups of respondents.

**Discussion.**

 The first sentence in the discussion is not supported by adequate statistical analysis. Furthermore, the authors need to elaborate on the main effects observed in this study before discussing other benefits of the intervention being tested.

**Conclusion**

 I also emphasize the research conclusions. The conclusion does not explicitly convey the final results of the research that has been conducted. The statement "The protocol of drinking warm water and Swedish abdominal massage immediately after waking up effectively reduces constipation scores on postoperative lower extremity fracture patients and can be used to adjuvant therapy" has not answered the aim of comparing the effectiveness of drinking warm water and Swedish abdominal massage on constipation scores on post-operative lower extremity fractures.

 Moreover, the recommendations given do not correspond to the research conclusions.

 Perhaps, the authors can improve this manuscript so that it can present clear research results and contain elements of novelty, described well and clearly, so that it can be acceptable and readable.

Is the work clearly and accurately presented and does it cite the current literature?

Partly

If applicable, is the statistical analysis and its interpretation appropriate?

Partly

Are all the source data underlying the results available to ensure full reproducibility?

Partly

Is the study design appropriate and is the work technically sound?

Yes

Are the conclusions drawn adequately supported by the results?

Partly

Are sufficient details of methods and analysis provided to allow replication by others?

Partly

Reviewer Expertise:

Cardiovascular Nursing

I confirm that I have read this submission and believe that I have an appropriate level of expertise to state that I do not consider it to be of an acceptable scientific standard, for reasons outlined above.

# Reviewer report: Reviewer response for version 2

**DOI:** 10.5256/f1000research.183692.r394054

**Published:** 2025-08-08

**Authors:** Nilton Carlos Machado

**Affiliations:** 1 São Paulo State University, São Paulo, Brazil

The authors accepted and did the correction solicited.

Is the work clearly and accurately presented and does it cite the current literature?

Yes

If applicable, is the statistical analysis and its interpretation appropriate?

Yes

Are all the source data underlying the results available to ensure full reproducibility?

Yes

Is the study design appropriate and is the work technically sound?

Yes

Are the conclusions drawn adequately supported by the results?

Yes

Are sufficient details of methods and analysis provided to allow replication by others?

Yes

Reviewer Expertise:

Pediatric Gastroenterology, Hepatology and Nutrition.

I confirm that I have read this submission and believe that I have an appropriate level of expertise to confirm that it is of an acceptable scientific standard.

# Reviewer report: Reviewer response for version 1

**DOI:** 10.5256/f1000research.174914.r357284

**Published:** 2025-02-11

**Authors:** Robin S Miccio

**Affiliations:** 1 Rutgers University, Newark, UK

Overall, this is a wonderful study. My biggest concern is with the strong statement in the conclusion: “The results showed that Swedish abdominal massage was noticeably more successful than warm water drinking therapy in reducing the constipation score.” The heterogeneity between groups at baseline makes this statement not entirely true. The authors note that “the statistical test found significant differences between the two intervention groups’ respondent characteristics (gender, age, and type of analgesic; p>0.05).” If the groups were significantly different at baseline, we are not comparing apples to apples. I’m a bit confused with p>0.05 – if p is greater than 0.05 that usually means that there is NO significant difference between groups? Would statisticians consider Levine’s Test to determine whether the assumption of homogeneity of variance is met? If there truly are differences at baseline, is another statistical test more appropriate to compare groups (like mixed-effects models)? If this is not possible, I think you can only compare pre and post data within groups (not between). It would also be good to note the limitations of a small sample size and report the effect size. Please note I am not a statistician, but thinking these areas need to be addressed.

 More information should be included about the groups. Such as: was the control group able to drink hot beverages during the intervention (i.e. tea or coffee), what about water consumption at baseline and throughout? Did the patients use laxatives at all? What other pain meds were used during the intervention (even non-opiate pain meds can cause constipation). From a massage therapist perspective, would be helpful to report details on the massage intervention – how was massage applied: over clothes/on skin, what techniques, what position was the patient in, who performed the massage and what was their training?

 Lastly, the way the aim is worded is confusing. I think the word “versus” or “against” may help clarify. It is also in the end of the Intro. Example correction: The objective of this study is to compare the effectiveness of drinking warm water versus Swedish abdominal massage on constipation scores on post-operative lower extremity fractures.

Is the work clearly and accurately presented and does it cite the current literature?

Yes

If applicable, is the statistical analysis and its interpretation appropriate?

Partly

Are all the source data underlying the results available to ensure full reproducibility?

No source data required

Is the study design appropriate and is the work technically sound?

Partly

Are the conclusions drawn adequately supported by the results?

Partly

Are sufficient details of methods and analysis provided to allow replication by others?

Partly

Reviewer Expertise:

Massage therapy

I confirm that I have read this submission and believe that I have an appropriate level of expertise to confirm that it is of an acceptable scientific standard, however I have significant reservations, as outlined above.

# Response

**Published:** 2025-06-14

**Authors:** Enny Selawaty Boangmanalu

**Affiliations:** Faculty of Nursing, Universitas Indonesia, Depok, West Java, Indonesia

Thank you for your feedback. We have reviewed and implemented your suggestions. All feedback has been addressed through revisions to the article.

 1. **Respon: **The respondent characteristics (gender, age, and type of analgesic; p>0.05) indicate that there was no significant difference between the two intervention groups—those receiving warm water therapy and those receiving Swedish abdominal massage. This conclusion is supported by the statistical tests (p-value > 0.05; α ≥ 0.05), which demonstrate no significant variance between the groups. The homogeneity of variance was tested using the chi-square test and Levene’s test, confirming that the groups were comparable at baseline.

 2. **Respon:** The homogeneity of respondent characteristics was tested using the chi-square test and Levine’s test. These tests confirmed that the assumption of homogeneity of variance was met, allowing for valid comparisons between the intervention groups. We acknowledge that alternative statistical methods, such as mixed-effects models, may provide further insight, particularly if baseline differences were present. Additionally, we recognize the importance of discussing the limitations of our sample size and reporting the effect size for a more comprehensive analysis.

 3. **Respon: **In the warm water therapy group, participants consumed 500 ml of warm water (temperature: approximately 31°C) each morning before breakfast. No other hot beverages (such as tea or coffee) were permitted during the intervention. Participants were allowed to eat breakfast 30-45 minutes after consuming the warm water.

 To minimize confounding factors, only non-opioid analgesics were used during the study, and no participants were given laxatives.

 The Swedish abdominal massage was performed directly on the skin, involving specific abdominal muscle movements. Each massage session lasted 15-20 minutes and was conducted daily for three consecutive days immediately after waking up. Patients remained in a supine position during the massage and were instructed to delay breakfast for 30-45 minutes post-intervention. The massages were administered by trained nurses, in accordance with their professional scope of practice under national nursing regulations.

**4. Respon: **The objective of this study is to compare the effectiveness of drinking warm water versus Swedish abdominal massage on constipation scores on post-operative lower extremity fractures. We accept your advice, we have revised our article

# Reviewer report: Reviewer response for version 1

**DOI:** 10.5256/f1000research.174914.r357292

**Published:** 2025-01-25

**Authors:** Nilton Carlos Machado

**Affiliations:** 1 São Paulo State University, São Paulo, Brazil

The authors present an enjoyable alternative for treating constipation. This study observed that its application was restricted to patients undergoing postoperative orthopedic surgery for lower extremity fractures (the femur, pelvis, tibia, fibula, ankle, and pedis).

 The study was elegantly designed and executed with great skill and accomplishment.

 The small number of patients is noteworthy. The term "Closed Fracture Reduction" does not fit well with the study. Perhaps a more fitting term could be suggested.

 Title. Please insert "postoperative orthopedic surgery" since the sample is restricted to this type of surgery.

 Please include the keywords in the abstract, title, and conclusions to increase the study's visibility.

 Results. Avoid repeating information in the text and Tables. In this manuscript, I think the variable "Gender" can be replaced by "Sex."

 Conclusions. Adequate e also concerning further studies.

Is the work clearly and accurately presented and does it cite the current literature?

Yes

If applicable, is the statistical analysis and its interpretation appropriate?

Yes

Are all the source data underlying the results available to ensure full reproducibility?

Yes

Is the study design appropriate and is the work technically sound?

Yes

Are the conclusions drawn adequately supported by the results?

Yes

Are sufficient details of methods and analysis provided to allow replication by others?

Yes

Reviewer Expertise:

Pediatric Gastroenterology, Hepatology and Nutrition.

I confirm that I have read this submission and believe that I have an appropriate level of expertise to confirm that it is of an acceptable scientific standard, however I have significant reservations, as outlined above.

# Response

**Published:** 2025-06-14

**Authors:** Enny Selawaty Boangmanalu

**Affiliations:** Faculty of Nursing, Universitas Indonesia, Depok, West Java, Indonesia

Thank you for your feedback. We have reviewed and implemented your suggestions. All feedback has been addressed through revisions to the article.

 1. The small number of patients is noteworthy. The term "Closed Fracture Reduction" does not fit well with the study. Perhaps a more fitting term could be suggested.

 Title. Please insert "postoperative orthopedic surgery" since the sample is restricted to this type of surgery.

**Response: **Thank you for your insightful feedback. I will revise the article accordingly and consider using a more appropriate term. I will also update the title to reflect the specific focus on postoperative orthopedic surgery.

 2. Please include the keywords in the abstract, title, and conclusions to increase the study's visibility.

**Response: **Thank you for your suggestion. We will ensure that the relevant keywords are incorporated into the abstract, title, and conclusions to enhance the study’s visibility.

 3. Results. Avoid repeating information in the text and Tables. In this manuscript, I think the variable "Gender" can be replaced by "Sex."

**Response: **Thank you for your comment. We will revise the manuscript to avoid redundancy between the text and tables, and we will replace "Gender" with "Sex" as recommended.

 4. Conclusions. Adequate e also concerning further studies.

**Response: **Thank you for the comment. We will revise the conclusion to better address this, emphasizing the importance of further research in this area.
